# Parental warmth buffers the negative impact of weaker fronto‐striatal connectivity on early adolescents' academic achievement

**DOI:** 10.1111/jora.12949

**Published:** 2024-05-08

**Authors:** Beiming Yang, Zexi Zhou, Ya‐Yun Chen, Varun Devakonda, Tianying Cai, Tae‐Ho Lee, Yang Qu

**Affiliations:** ^1^ School of Education and Social Policy Northwestern University Evanston Illinois USA; ^2^ Department of Human Development and Family Sciences The University of Texas at Austin Austin Texas USA; ^3^ Department of Psychology Virginia Tech Blacksburg Virginia USA; ^4^ Institute of Child Development University of Minnesota, Twin Cities Minneapolis Minnesota United States

**Keywords:** academic achievement, adolescence, frontoparietal, inhibitory control, parental warmth, striatum

## Abstract

In past decades, the positive role of self‐control in students' academic success has attracted plenty of scholarly attention. However, fewer studies have examined the link between adolescents' neural development of the inhibitory control system and their academic achievement, especially using a longitudinal approach. Moreover, less is known about the role of parents in this link. Using large‐scale longitudinal data from the Adolescent Brain Cognitive Development (ABCD) study (*N* = 9574; mean age = 9.94 years at baseline, SD = .63; 50% girls), the current study took an integrative biopsychosocial approach to explore the longitudinal link between early adolescents' fronto‐striatal connectivity and their academic achievement, with attention to the moderating role of parental warmth. Results showed that weaker intrinsic connectivity between the frontoparietal network and the striatum was associated with early adolescents' worse academic achievement over 2 years during early adolescence. Notably, parental warmth moderated the association between fronto‐striatal connectivity and academic achievement, such that weaker fronto‐striatal connectivity was only predictive of worse academic achievement among early adolescents who experienced low levels of parental warmth. Taken together, the findings demonstrate weaker fronto‐striatal connectivity as a risk factor for early adolescents' academic development and highlight parental warmth as a protective factor for academic development among those with weaker connectivity within the inhibitory control system.

## INTRODUCTION

In past decades, the positive role of self‐control in adolescents' academic achievement has attracted plenty of scholarly attention (Duckworth et al., 2014). It is not surprising that self‐control contributes to academic success. Basic tasks such as paying attention in class and completing homework assignments are not always easy. In general, compared to leisure and daily chores, learning is more difficult and frustrating (Bjork & Bjork, 2011). Although adolescents understand the importance of academic work, they also find it less enjoyable compared to other activities (Duckworth et al., 2019). Therefore, it requires self‐control for adolescents to inhibit the temptation of more enjoyable activities to focus on the more important academic work. Indeed, self‐control has been found to be consistently associated with greater academic attainment, course grades, and standardized test scores (e.g., Galla et al., 2014; Moffitt et al., 2011; Richardson et al., 2012). However, past studies on adolescents' self‐control mainly used self‐ or parent‐reported measures and behavioral assessments (Duckworth & Kern, 2011).

Recently, a growing body of neuroscientific research has shown that neural activation in several brain regions, namely the inhibitory control system, is associated with greater self‐control ability, leading to better behavioral and academic adjustment during adolescence (e.g., Chen et al., 2023; da Costa et al., 2022; DePasque & Galván, 2017; Jolles et al., 2020; Lee & Telzer, 2016; Somerville et al., 2011; Van Den Bos et al., 2015). However, previous brain studies have mainly focused on cross‐sectional samples to examine the relationship between the inhibitory control system and academic achievement in adolescence, despite the fact that adolescence is a period of significant changes in learning behaviors (e.g., decreased academic engagement; Wang & Eccles, 2012). Therefore, it is crucial to employ longitudinal designs to investigate the role of the inhibitory control system in academic achievement over adolescence. Moreover, despite the important role of parents in adolescents' self‐control processes (Cullen et al., 2008), less is known about the role of parents in the link between adolescents' inhibitory control system and their academic achievement. These gaps in the literature call for research that bridges neuroscience, educational science, and family science to provide a more comprehensive understanding of the neural basis of inhibitory control in academic success. Advances in this line of research will provide insights into how and when the developing brain matters for learning. Therefore, using large‐scale longitudinal data from the Adolescent Brain Cognitive Development (ABCD) study, the current study took a biopsychosocial approach to explore the longitudinal link between early adolescents' fronto‐striatal connectivity and their academic achievement, with attention to the moderating role of parental warmth (i.e., acceptance and affection from parents).

Prior research has linked low self‐control to a failure of the brain's inhibitory control processes, derived from the neurodevelopmental imbalance between the top‐down executive regions and bottom‐up subcortical regions (Brand et al., 2014; Casey, 2015; Lee & Telzer, 2016). In particular, functional connectivity between regions in the frontoparietal network (FPN) and striatum has been highlighted as an indicator of the neural maturation level of inhibitory control (Chen et al., 2023; Rubia et al., 2006). The FPN, primarily consisting of the dorsolateral prefrontal cortex (DLPFC) and the posterior parietal cortex, is a neural network that is key to problem‐solving and sustained attention (Marek & Dosenbach, 2018). The striatum, which consists of the caudate, putamen, and nucleus accumbens (NAcc), plays a key role in decision‐making and reward processing (Valjent & Gangarossa, 2021). Instead of focusing on a single region involved in inhibitory control processes, recent research suggested that it would be more comprehensive to examine inhibitory control processes with a circuit‐based approach to view between‐network connectivity as a system (Casey, 2015; Hampshire & Sharp, 2015). At the brain‐systems level, the FPN and the striatum are both structurally and functionally connected, which forms part of the fronto‐striatal circuit that supports inhibitory control through the DLPFC (Haber, 2016; Leh et al., 2007; Zhang & Iwaki, 2020). Importantly, neural activations in the fronto‐striatal circuit increase along with inhibition efficiency across adolescence and adulthood (Rubia et al., 2006), which suggests that the connectivity between the FPN and the striatum is an indicator of the maturation of inhibitory control. Given the importance of self‐control in adolescents' academic success, it is likely that adolescents with weaker fronto‐striatal connectivity may struggle academically over time.

Although adolescence is a period where children increasingly seek individuation from parents (Koepke & Denissen, 2012), parents still exhibit large influences on their development (Hoskins, 2014; Morris et al., 2021). Therefore, it is crucial to examine the role of parents in the link between adolescents' fronto‐striatal connectivity and their academic achievement. Parental warmth may buffer the potential negative effect of weaker fronto‐striatal connectivity on adolescents' academic achievement. Past studies on behavioral development found that parental support and positive parent–child relationship weaken the links between adolescents' lack of self‐control and their greater behavioral problems (Higgins & Boyd, 2008; Jones et al., 2007; Liu et al., 2019). The same may apply to the relationship between fronto‐striatal connectivity and academic development. Although weaker fronto‐striatal connectivity may represent inadequate ability to employ the neural resources to support self‐control (Hampshire & Sharp, 2015; Rubia et al., 2006), adolescents with fronto‐striatal connectivity do not necessarily lack the intention to work hard in school. For adolescents who want to control their impulses but lack the efficacy to do so, parental warmth (i.e., acceptance and affection from parents) may be particularly helpful in setting them on the right path. For example, when adolescents experience greater warmth from parents, they can seek help from parents for extra motivation to focus on academic work. Similarly, when adolescents experience high acceptance from parents, executing self‐control to work hard in school may be more rewarding. Overall, by showing warmth to adolescents, parents may cultivate a positive environment that is easier for adolescents to engage in self‐control even if they have weaker fronto‐striatal connectivity.

## THE PRESENT STUDY

Using data from the ABCD study, the current study took a biopsychosocial approach to examine the role of FPN‐striatum connectivity in academic achievement (i.e., school grades and parent‐perceived school performance) over 2 years in early adolescence, with attention to the moderating role of parental warmth. Guided by prior research, we had two hypotheses. First, we hypothesized that weaker FPN‐striatum connectivity would predict early adolescents' decreased school grades and parent‐perceived school performance 2 years later. Second, we hypothesized that parental warmth would moderate the longitudinal links between FPN‐striatum connectivity and school grades/performance, such that FPN‐striatum connectivity would only have a significant effect on school grades/performance among early adolescents who reported low warmth from parents.

## METHODS

### Participants

Data were obtained from baseline (T1) and 2‐year follow‐up (T2) of the ABCD study (data release 5.0). All the data included in the current study are available on the NIMH Data Archive (https://nda.nih.gov/abcd) upon data access request. Participants of the ABCD study were recruited at 21 sites in the United States using probability sampling (Garavan et al., 2018). Previous work documents a variety of measures that were used for this study, including task‐based fMRI and behavioral outcomes (Casey et al., 2018). A total of 9574 early adolescents (mean age = 9.94 years, SD = .63; 50% girls) and their parents (primary caregivers of adolescents; 89% mothers) were included in the analyses. The current research included subjects based on the inclusion criteria provided by the ABCD team (i.e., participants with variable “imgincl_rsfmri_include” = 1), which are the recommended quality control criteria of resting‐state fMRI in ABCD data release note 5.0 (e.g., FreeSurfer quality control and fMRI manual post‐processing quality control; for detailed criteria, see ABCD Human Subjects Study, 2023).

### Resting‐state fMRI data acquisition and preprocessing

Resting‐state fMRI data were acquired at T1. During the 20‐min resting‐state data acquisition, adolescents were instructed to keep their eyes open and passively look at a fixation cross at the center of the screen. The ABCD study used three 3 T scanner platforms (i.e., Siemens Prisma [Siemens Healthineers], GE 750 [GE Healthcare], and Philips [Philips Healthcare]) with a harmonized neuroimaging protocol across 21 sites. Each scanner used a standard head coil for the initial time point of fMRI data acquisition. Prior to the MRI scanning sessions, adolescents practiced motion compliance in a simulated MRI environment with motion‐capture devices that provided feedback to the adolescent. Images were preprocessed with motion correction, b0 distortion correction, isotropic resampling, structural image coregistration, and spatial normalization. AFNI's 3dvolreg (Cox, 1996) was used to correct for head motion. Distortions were also addressed by reversing the polarity of the signal (Holland et al., 2010). The displacement field was computed using several spin‐echo calibration scans, adjusted using estimates for between‐scan head motion, and then applied to the sequence of gradient‐echo images to prevent signal “drop‐out” caused by within‐voxel field gradients in gradient‐echo images. The distortions caused by gradient nonlinearities were then removed from the images (Jovicich et al., 2006). Reference scans were chosen for each participant to help correct between‐scan motion. The initial frame from each scan was rigidly aligned with the first frame of the reference scan, and automated registration between spin‐echo, calibration scans, and structural images was carried out using mutual information with coarse pre‐alignment (Hagler Jr et al., 2019). The ABCD team applied several steps to remove subjects with poor imaging quality. First, subjects were excluded according to automated quality control metrics of mean motion, the number of seconds with framewise displacements <0.2, 0.3, or 0.4 mm (Power et al., 2012), and temporal SNR (tSNR) (Triantafyllou et al., 2005). Then, the ABCD team manually inspected for signs of artifacts and poor image quality and manually reviewed FreeSurfer's cortical surface reconstruction (Fischl, 2012) to gauge the severity of artifacts or processing problems. For more information on data acquisition and preprocessing of the ABCD study, see Casey et al. (2018) and Hagler Jr et al. (2019).

### Intrinsic connectivity between the frontoparietal network and the striatum

**FIGURE 1:**
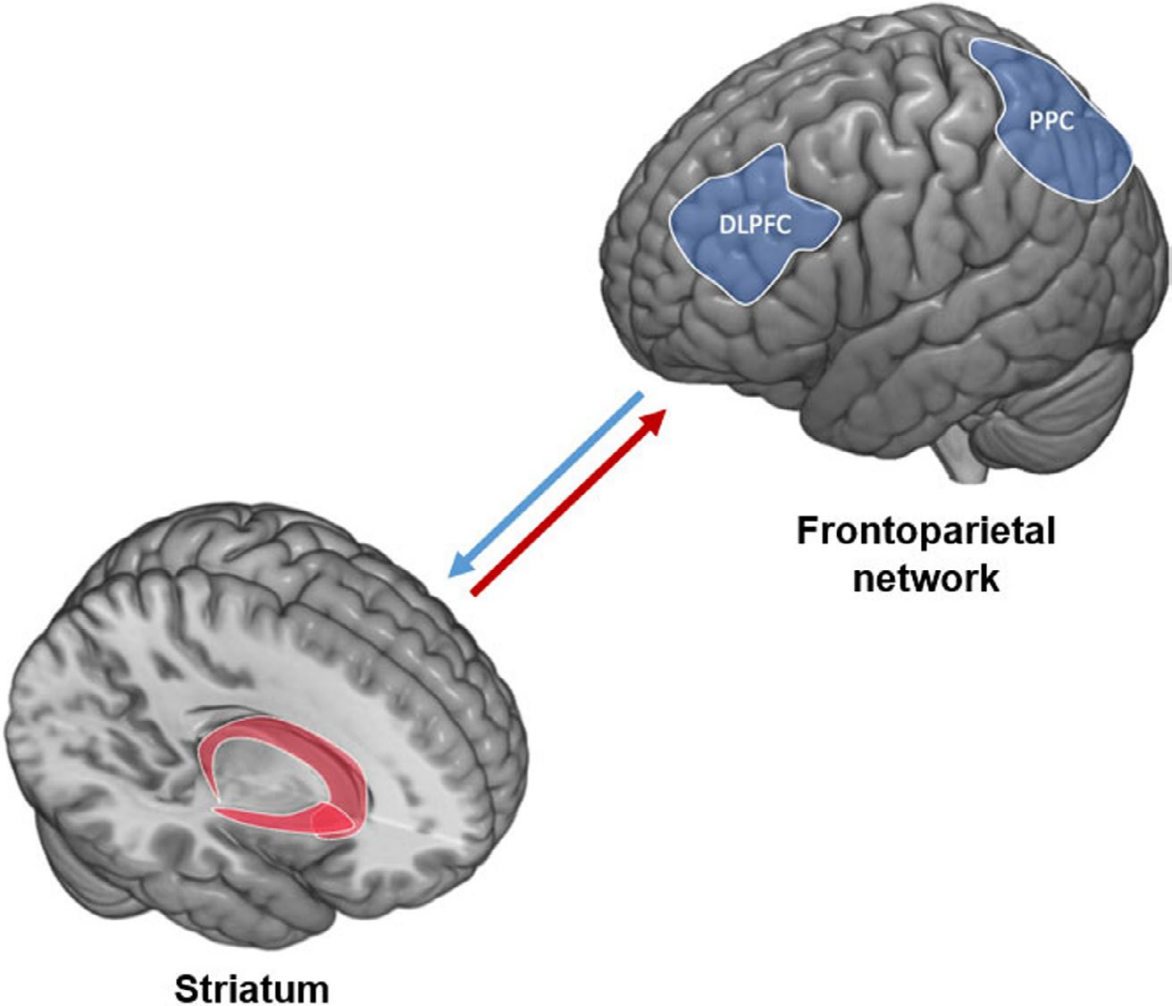
Illustration of the connectivity between the frontoparietal network and the striatum.

The ABCD dataset provides processed data on the strengths of the connectivity between cortical networks and subcortical regions. Cortical networks were extracted using the Gordon parcellation approach (Gordon et al., 2016), and subcortical regions were derived using FreeSurfer's automated brain segmentation (aseg) atlas (Fischl, 2012). For more details on MRI processing pipeline and ROI extraction used in the ABCD study, see Hagler Jr et al. (2019). Given the key role of the connectivity between frontoparietal regions and striatal regions in inhibitory control (Casey, 2015; Chen et al., 2023; Rubia et al., 2006), the current study focused on the strength of the FPN to striatum connectivity (as shown in Figure 1). Following the practice of prior research that examined this connectivity using the ABCD study data (Chen et al., 2023), FPN to striatum connectivity was calculated by averaging the connectivity of FPN to bilateral caudate, FPN to bilateral putamen, and FPN to bilateral accumbens.

### Questionnaire measures

#### Adolescents' academic achievement

At T1 and T2, parents reported on their children's average letter grades during the past year (1 = *A*, 2 = *B*, 3 = *C*, 4 = *D*, 5 = *F*). The score was reversed, with a higher number indicating higher school grades. At T1 and T2, on a 4‐point scale (1 = *very well*, 2 = *average*, 3 = *below average*, 4 = *failing*), parents also reported on how well they thought their children were doing in school during the past year. The score was reversed, with a higher number indicating better school performance perceived by parents. These measures of parent‐reported academic achievement have been widely used in past studies using the ABCD study data (e.g., Clark et al., 2021; Paulich et al., 2021; Tomasi & Volkow, 2021).

#### Parental warmth

At T1, parental warmth was assessed using the acceptance subscale of the Child Report of Behavior Inventory (CRPBI; Schaefer, 1965), which consists of five items measuring adolescents' perceptions of their parents' warmth (e.g., “believe in showing love for me” and “smile at me very often”). Adolescents indicated the extent to which they agreed with each item on a 3‐point Likert scale (1 = *not at all* to 3 = *very much*). This measure showed adequate internal consistency, with McDonald's Omega (ω) = .71. The average was taken across all five items, with a higher number indicating greater parental warmth.

#### Demographic characteristics

In line with past studies using the resting‐state data of the ABCD study (Brooks et al., 2021; Umbach & Tottenham, 2021), the current study included adolescents' age, biological sex, race, and family income as demographic covariates. Each of these demographic characteristics was found to be associated with adolescents' academic achievement (Brass et al., 2019; Caro et al., 2009). Adolescents' age was their age at the baseline assessment. Adolescents' biological sex was coded into 0 = male and 1 = female. Adolescents' race was coded into 0 = White and 1 = racial minority. Family income was the annual income ranged from 1 = <$5000 to 10 = $200,000 and greater.

### Overview of the analyses

Two sets of analyses were conducted to test the hypotheses using Mplus 8.9 (Muthén & Muthén, 2023). The attrition rate from T1 to T2 was 9.8%. Besides the attrition from T1 to T2, there were 0.02% missing data in adolescents' biological sex and 8.3% missing data in family income. Little's MCAR test suggested that the data were not missing completely at random (*X* ^2^ = 719.32, df = 254, *p* < .001; Little, 1988). Participants who completed both waves were then compared to participants who completed only the first wave to examine whether the missing data were conditioned on other observed variables. Results showed that participants who completed both waves had higher family income, lower percentage of racial minorities, higher grades, and higher subjective academic performance (*p*s < .001). This pattern of data missing suggests that it was missing at random (MAR), related to observed but not unobserved data (Mack et al., 2018). Therefore, maximum likelihood estimation with robust standard errors (MLR), which is an estimator robust to non‐normality and non‐independence (Kline, 2015), was used to handle missing data and provide unbiased standard errors. Demographic variables and baseline academic performance were included in all the models to ensure the robustness of the missing data assumption. To account for the nested structure of the sampling with siblings within a family, the Taylor series linearization using the TYPE = COMPLEX command in Mplus was applied to all models. As for the clustering effect derived from the multisite design, the STRATIFICATION = SITE ID command in Mplus was used to take into account the non‐independence of the observations.

The first set of analyses examined the main effect of adolescents' FPN‐striatum connectivity on their school grades and parent‐perceived school performance over time. In the same model, school grades and parent‐perceived school performance at T2 were predicted by FPN‐striatum connectivity at T1, controlling for school grades (or parent‐perceived school performance) at T1 and demographic covariates (i.e., adolescents' age, biological sex, race, and family income). The second set of analyses tested the moderating role of parental warmth in the longitudinal associations between FPN‐striatum connectivity and academic achievement (i.e., school grades and parent‐perceived school performance). In the same model, school grades and parent‐perceived school performance at T2 were predicted by FPN‐striatum connectivity at T1, parental warmth at T1, and FPN‐striatum connectivity × parental warmth at T1, controlling for school grades (or parent‐perceived school performance) at T1 and demographic covariates. For moderation analyses, predictors and moderators were mean‐centered before entering the model. The interaction effects were then probed using the simple slope technique (Bauer & Curran, 2005), which estimates and presents the associations between FPN‐striatum connectivity and school grades/performance among adolescents with a low level (i.e., 1 SD below the mean) and a high level (i.e., 1 SD above the mean) of parental warmth. All models were saturated with perfect fit (CFIs = 1.00, TLIs = 1.00, SRMRs = .00, RMSEAs = .00), such that all possible links were included.

## RESULTS

### Descriptive statistics and bivariate correlations

Table 1 shows the descriptive statistics and bivariate correlations between the variables included in the current study. FPN‐striatum connectivity was positively associated with school grades and parent‐perceived school performance both concurrently and 2 years later. Parental warmth was also positively associated with school grades and parent‐perceived school performance at both timepoints. However, FPN‐striatum connectivity was not associated with parental warmth. Girls had weaker FPN‐striatum connectivity than boys. At the same time, girls reported higher parental warmth and had better school grades and parent‐perceived school performance. Racial minorities had weaker FPN‐striatum connectivity and worse school grades and parent‐perceived school performance. Financial adversity was negatively associated with FPN‐striatum connectivity, school grades, and parent‐perceived school performance.

**TABLE 1:** Descriptive statistics and correlations of variables.

	1	2	3	4	5	6	7	8	9	10
1. T1 FPN‐striatum connectivity	–									
3. T1 parental warmth	.01	–								
4. T1 school grades	.06***	.07***	–							
5. T1 parent‐perceived school performance	.05***	.07***	.76***	–						
6. T2 school grades	.06***	.08***	.59***	.51***	–					
7. T2 parent‐perceived school performance	.05***	.09***	.54***	.54***	.79***	–				
8. Adolescents' age	−.02*	.01	−.03**	−.02*	−.04***	−.03*	–			
9. Adolescents' biological sex	−.07***	.06***	.10***	.10***	.12***	.11***	−.03***	–		
10. Adolescents' race	−.13***	−.04***	−.23***	−.15***	−.21***	−.14***	−.03**	.03*	–	
11. Family income	.11***	.06***	.30***	.21***	.31***	.21***	.04***	−.00	−.43***	–
Mean	.02	2.79	4.36	3.59	4.36	3.57	9.94	.50	.46	7.34
SD	.03	.30	.77	.60	.80	.61	.63	.50	.50	2.34
Min	−.11	1	1	1	1	1	8.92	0	0	1
Max	.16	3	5	4	5	4	11.08	1	1	10

*Note*: For adolescents' biological sex, 0 = male and 1 = female; for adolescents' race, 0 = White and 1 = minority; family income ranged from 1 (<$5000) to 10 ($200,000 and greater). *p < .05; **p < .01; ***p < .001.

Abbreviation: FPN, frontoparietal network.

### FPN‐striatum connectivity and academic achievement

**FIGURE 2:**
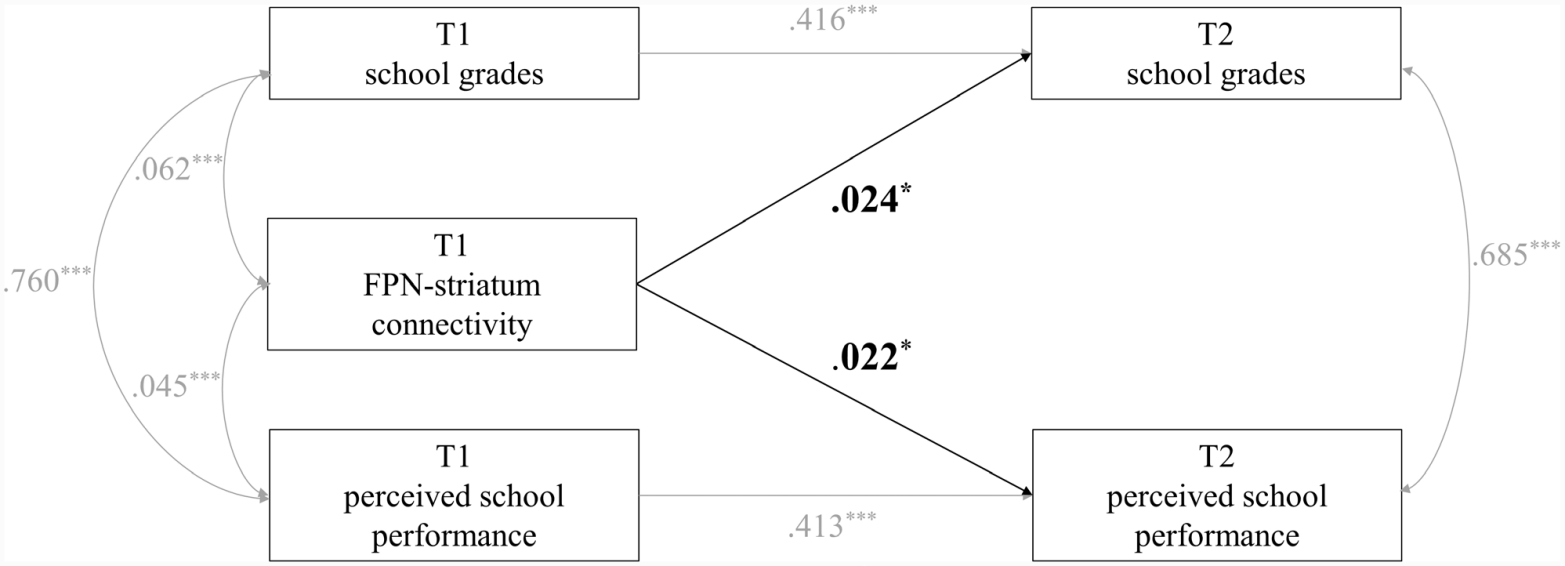
Predicting school grades and parent‐perceived school performance over time from FPN‐striatum connectivity. FPN, frontoparietal network. Demographic covariates (i.e., adolescents' age, biological sex, race, and family income) were controlled in the analyses but were not pictured for ease of presentation. Standardized coefficients were presented. *p < .05, ***p < .001.

In the first set of analyses, adolescents' school grades and parent‐perceived school performance at T2 were predicted by FPN‐striatum connectivity at T1, grades/performance at T1, and demographic covariates (i.e., adolescents' age, biological sex, race, and family income). As shown in Figure 2 and Table 2, FPN‐striatum connectivity was associated with increased school grades over 2 years during early adolescence (*β* = .024, *p* = .011), adjusting for prior school grades and demographic covariates. Similarly, FPN‐striatum connectivity was associated with increased parent‐perceived school performance over time (*β* = .022, *p* = .027), adjusting for prior school performance and demographic covariates.

**TABLE 2:** Predicting school grades and parent‐perceived school performance over time from FPN‐striatum connectivity.

	Predicting school grades at T2			Predicting parent‐perceived school performance at T2		
	*B*	SE	*β*	*B*	SE	*β*
Adolescents' age	−.030	.012	−.024*	−.018	.009	−.019*
Adolescents' biological sex	.134	.015	.087***	.090	.012	.077***
Adolescents' race	−.079	.017	−.051***	−.037	.013	−.032**
Family income	.060	.005	.185***	.033	.003	.131***
Prior school grades/performance	.410	.012	.416***	.407	.012	.413***
FPN‐striatum connectivity	.635	.251	.024*	.442	.199	.022*

*Note*: For adolescents' biological sex, 0 = male and 1 = female; for adolescents' race, 0 = White and 1 = minority; family income ranged from 1 (<$5000) to 10 ($200,000 and greater). *p < .05; **p < .01; ***p < .001.

Abbreviation: FPN, frontoparietal network.

### The moderating role of parental warmth

**FIGURE 3:**
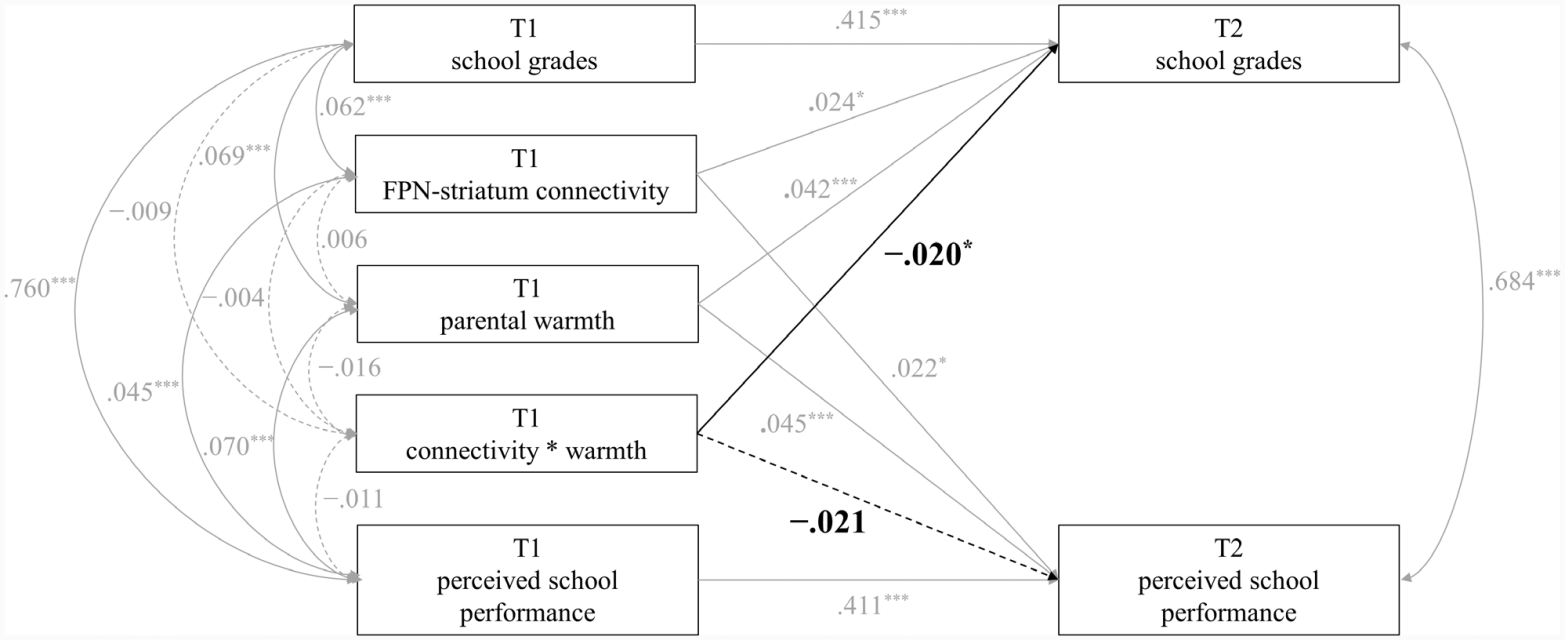
Parental warmth moderated the longitudinal associations between FPN‐striatum connectivity and school grades/parent‐perceived school performance. FPN, frontoparietal network. Demographic covariates (i.e., adolescents' age, biological sex, race, and family income) were controlled in the analyses but were not pictured for ease of presentation. Standardized coefficients were presented. *p < .05, ***p < .001.

**FIGURE 4:**
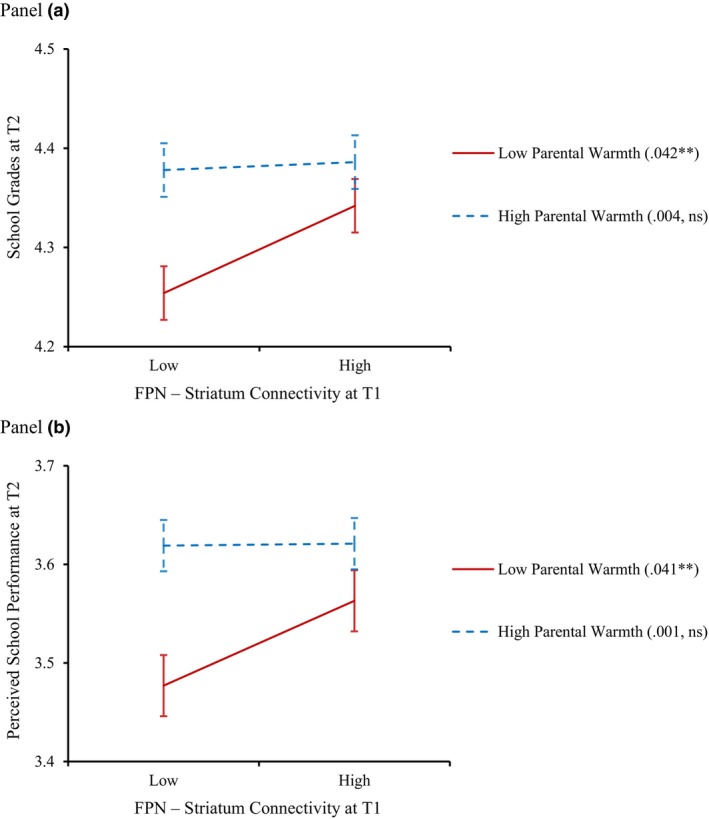
Simple slopes of the association between FPN‐striatum connectivity and school grades (Panel a)/parent‐perceived school performance (Panel b) at low and high parental warmth. FPN, frontoparietal network. School grades/performance at T1 and demographic covariates were controlled in the analyses. Low (or high) parental warmth is 1 SD below (or above) the mean. Standardized simple slopes are presented in parentheses. **p < .01; ns, not significant.

The second set of analyses examined whether parental warmth moderated the longitudinal associations between adolescents' FPN‐striatum connectivity and academic achievement (i.e., school grades and parent‐perceived school performance), controlling for prior school grades/performance and demographic covariates. As shown in Figure 3 and Table 3, parental warmth moderated the longitudinal association between FPN‐striatum connectivity and school grades (interaction term: *β* = −.020, *p* = .043). The moderating effect of parental warmth in the longitudinal association between FPN‐striatum connectivity and parent‐perceived school performance was not significant (interaction term: *β* = −.021, *p* = .070); nevertheless, this moderating effect was in the same direction and has a comparable standardized coefficient as the moderating effect regarding the link between FPN‐striatum connectivity and school grades. Simple slopes of the longitudinal effects of FPN‐striatum connectivity on school grades and parent‐perceived school performance were plotted for adolescents who reported low parental warmth (i.e., 1 SD below the mean) and high parental warmth (i.e., 1 SD above the mean). As shown in Panel A of Figure 4, the longitudinal association between FPN‐striatum connectivity and school grades was significant when parental warmth was low (*β* = .042, *p* = .002), but not when parental warmth was high (*β* = .004, *p* = .732). Similarly, as shown in Panel B of Figure 4, the longitudinal association between FPN‐striatum connectivity and parent‐perceived school performance was significant when parental warmth was low (*β* = .041, *p* = .009) but not when parental warmth was high (*β* = .001, *p* = .963).

**TABLE 3:** Positive parenting moderated the longitudinal associations between FPN‐striatum connectivity and school grades/performance.

	Predicting school grades at T2			Predicting parent‐perceived school performance at T2		
	*B*	SE	*β*	*B*	SE	*β*
Adolescents' age	−.030	.012	−.024*	−.019	.009	−.020*
Adolescents' biological sex	.131	.015	.086***	.088	.012	.075***
Adolescents' race	−.079	.017	−.051***	−.037	.013	−.032**
Family income	.060	.005	.182***	.032	.003	.128***
Prior school grades/performance	.409	.012	.415***	.406	.012	.411***
FPN‐striatum connectivity	.064	.025	.024**	.044	.020	.022*
Parental warmth	.109	.027	.042***	.099	.022	.050***
FPN‐striatum connectivity × Parental warmth	−.175	.086	−.020*	−.145	.080	−.021

*Note*: FPN‐striatum connectivity was multiplied by 10 for the model's identifiability. For adolescents' biological sex, 0 = male and 1 = female; for adolescents' race, 0 = White and 1 = minority; family income ranged from 1 (<$5000) to 10 ($200,000 and greater). *p < .05; **p < .01; ***p < .001.

Abbreviation: FPN, frontoparietal network.

## DISCUSSION

Despite plenty of scholarly attention on self‐control and academic success (Duckworth et al., 2019), it is less clear about how adolescents' brain profiles related to inhibitory control may play a role in their academic achievement over time. Using large‐scale longitudinal data, the results of the current study suggest that weaker intrinsic connectivity between the FPN and the striatum is a risk factor for early adolescents' academic development. Importantly, such longitudinal links were only significant when early adolescents experienced low levels of parental warmth, which highlights parental warmth as a protective factor for academic development among early adolescents with weaker FPN‐striatum connectivity.

In line with past research on the key role of self‐control in adolescents' academic achievement (Galla et al., 2014; Moffitt et al., 2011; Richardson et al., 2012), our results showed that, as a possible indicator of an immature inhibitory control system, weaker fronto‐striatal connectivity was associated with worse school grades and parent‐perceived school performance over 2 years in early adolescence. Fronto‐striatal connectivity is certainly only one factor in self‐control. Nevertheless, this indicator of inhibitory control system does play an important role in early adolescents' academic achievement. Early adolescents with weaker fronto‐striatal connectivity may be less capable of recruiting the neural circuit to support inhibitory control when needed. In this case, it may be more difficult for them to successfully inhibit the impulse of doing other activities (e.g., checking social media) when they need to focus on academic work. The finding supports and extends the current knowledge on fronto‐striatal connectivity as a marker of the maturation of the inhibitory control system (Casey, 2015; Chen et al., 2023; Rubia et al., 2006). To the best of our knowledge, the current study is the first to explore the longitudinal role of fronto‐striatal connectivity in early adolescents' academic development. It is important for future studies to examine whether fronto‐striatal connectivity also contributes to other aspects of adolescent development.

Notably, parental warmth moderated the association between fronto‐striatal connectivity and academic achievement. Among early adolescents who experienced lower levels of warmth from parents, weaker fronto‐striatal connectivity had a larger negative effect on their academic achievement. In contrast, among early adolescents who experienced higher levels of warmth from parents, weaker fronto‐striatal connectivity did not have a negative effect on their academic achievement. These results are consistent with prior research on how parental support buffers the negative impact of low self‐control on adolescent behavioral development (e.g., deviant and risk‐taking behaviors; Higgins & Boyd, 2008; Jones et al., 2007; Liu et al., 2019).

Adolescents may feel more rewarded for successfully executing self‐control when they live in an environment with high parental warmth. Parental warmth may help cultivate a supportive family environment for adolescents, making it easier for adolescents to engage in self‐control regardless of their fronto‐striatal connectivity. Moreover, adolescents may rely less on their inhibitory control if their parents have helped them develop enough motivation for academics. For example, parental advising and family rules can improve adolescents' intrinsic motivation toward learning (Fan & Williams, 2010). Similarly, parental involvement in learning promotes adolescents' parent‐oriented motivation, which in turn contributes to their academic success (Cheung & Pomerantz, 2012). Future studies should examine how different dimensions of parental involvement alter the impact of fronto‐striatal connectivity on adolescents' academic achievement. Overall, the finding is encouraging as it shows that the negative impact of weaker fronto‐striatal connectivity on academic achievement can be buffered by parental warmth. Therefore, parents do not need to be overly worried about the aversive effect of weaker fronto‐striatal connectivity on academic achievement because they can neutralize the negative effects by showing love to adolescents, which are feasible strategies for most parents.

### Interdisciplinarity and implications for preventive interventions

A strength of the current study is the interdisciplinary approach. Conceptually, this study integrated neuroscience (i.e., inhibitory control system), educational science (i.e., academic achievement over 2 years), and family science (i.e., parenting practices). Methodologically, the current research included both neuroimaging and survey assessments using rigorous longitudinal analyses. This interdisciplinary perspective on the role of early adolescents' fronto‐striatal connectivity in their academic development as well as the protective role of parental warmth provides two key insights for preventive intervention. First, this study identified weaker fronto‐striatal connectivity as a risk factor for early adolescents' academic development. School interventions that emphasize self‐control in learning (e.g., self‐regulated learning interventions) should take into consideration individual differences in students' inhibitory control system that may influence the effects of such interventions. Moreover, this highlights the importance of further research on risk factors for adolescents' inhibitory control system. Future interventions targeting risk factors for the development of inhibitory control system may positively contribute to adolescents' academic development. Second, by identifying the moderating role of parental warmth in the link between fronto‐striatal connectivity and academic achievement, the current study suggests that parental warmth may be an effective direction for interventions in promoting early adolescents' flourishing among those with weaker fronto‐striatal connectivity. At the same time, the findings point to the importance of taking into consideration early adolescents' brain development when implementing family‐based interventions.

### Limitations and future directions

There are a few limitations in the present study that point to directions for future research. First, despite the use of longitudinal methods, the findings are correlational in nature, and thus the causality should be interpreted with caution. Second, the effect sizes of the moderation findings are very small. The small effect size is expected because prior studies using the ABCD study data also found comparable effect sizes of brain × environment interactions (Gunther et al., 2023; Liu et al., 2021; Yang et al., 2023). This suggests that brain × environment interactions among early adolescents are likely to only have small impacts on adolescent adjustment. In this case, practical implications of the moderation findings should be taken with caution.

Third, although fronto‐striatal connectivity has been suggested as an indicator of the maturation of the inhibitory control system (e.g., Chen et al., 2023; DePasque & Galván, 2017; Rubia et al., 2006), it also serves many other functions (Leh et al., 2007). At the same time, there are many other neural activities that are relevant for inhibitory control (da Costa et al., 2022; Jolles et al., 2020). Therefore, future studies should examine additional brain profiles related to inhibitory control to further elucidate the role of the inhibitory control system in adolescents' academic development.

Fourth, the current study only examined one dimension of parenting behaviors (i.e., parental warmth) because the ABCD study only had very few parenting measures (Gonzalez et al., 2021) and parental warmth is the only one with satisfactory reliability. Future research should examine the role of other aspects of parenting (e.g., parenting involvement in learning) in the link between fronto‐striatal connectivity and academic achievement. Similarly, academic achievement was assessed based on parent‐reported grades and performance, because school records are not available. Future studies should explore the role of fronto‐striatal connectivity in academic achievement with more reliable measures such as course grades from school records.

Finally, the current study focused on early adolescents from 10 to 12 years old, which leaves an open question about whether the role of fronto‐striatal connectivity in academic achievement strengthens or weakens when adolescents enter mid and late adolescence. During mid and late adolescence, coursework may become more challenging, and adolescents' inhibitory control system may also become more developed. Future studies can follow adolescents over a longer period of time to examine the effects of fronto‐striatal connectivity on academic achievement over mid and late adolescence.

## CONCLUSIONS

Given that early adolescence is a period marked by significant neural changes as well as changes in academic functioning, it is important to explore the role of the adolescent brain in academic development. Using a large‐scale longitudinal sample of early adolescents, our results suggest that weaker intrinsic connectivity between the FPN and the striatum is associated with adolescents' worse academic achievement over time. Importantly, parental warmth can buffer the negative effect of weaker fronto‐striatal connectivity on adolescents' academic achievement. Our findings demonstrate weaker fronto‐striatal connectivity as a risk factor for early adolescents' academic development and also highlight parental warmth as a protective factor for academic development among adolescents with weaker connectivity within the inhibitory control system.

## FUNDING INFORMATION

This research is supported by the National Science Foundation (BCS‐1944644) and research fund from the Center for Culture, Brain, Biology, and Learning at Northwestern University to Yang Qu.

## CONFLICT OF INTEREST STATEMENT

The authors have no conflict of interest to declare.

## CONSENT FOR PUBLICATION

All authors consent for the publication of this manuscript.

## PARTICIPANT CONSENT STATEMENT

In the ABCD study, all participants have provided consent and assent to express their willingness to participate. All consenting participants (parents of the children who are the study subjects) have also provided consent to broad data sharing, including secondary data analyses beyond the prescribed endpoints of the ABCD study.

## Data Availability

Data were obtained from baseline and 2‐year follow‐up of the Adolescent Brain Cognitive Development (ABCD) study (data release 5.0). All the data included in the current study are available on the NIMH Data Archive (https://nda.nih.gov/abcd) upon data access request.
